# Supplementary material for: Chromothripsis during telomere crisis is independent of NHEJ, and consistent with a replicative origin
Source: Genome Res. 2019 May;29(5):737–49. doi: 10.1101/gr.240705.118 (PMC6499312; doi:10.1101/gr.240705.118)
Supplement: Supplemental Material [file supp_gr.240705.118_Supplemental_file_1.zip › contigs/annotated_contigs/DB54/contig.3.DB54_length_269_mean_cov_2.82527881041.docx]

**DB54_length_269_mean_cov_2.82527881041**

```
ATTTTATCAACGTAACTTGACATATGTTTTACAAGTCCATAATC|TCAGAGACTTATTTTTTTTTTTTTTTTTTTTTGA|G|GCTGGAG
                                             >chr17:65752850-65752885 + E=1e-03 >chr6:
TGCCATGGCACAATCTTGGCTCACTGCAGCCTTGACCTCCTGGACTC|GAG|ATCCTCCCACCTCAGCCTCTCAAGGAGCTGAGACCAC
21232281-21232336 + E=9e-16                        >chr14:20671322-20671399 + E=5e-26
AGGCACATGCCACCATGCCTGGCTAATTTTTTTTTTTTTT|CAGGGTGCGGGTCTTGCCATGTTTGCCAGGCTATTGGGTTGAAGGGGT

ACTCTCGA
```
